# Supplementary material for: Are carbon emissions trading and green financial instruments synergistic? -Comprehensive quantitative research based on content analysis
Source: PLoS One. 2024 Mar 7;19(3):e0298601. doi: 10.1371/journal.pone.0298601 (PMC10919601; doi:10.1371/journal.pone.0298601)
Supplement: S1 Data — (ZIP) [file pone.0298601.s001.zip › Data/地级市Z_F工作报告.zip!/║╙─╧/╓▄┐┌╩╨/└┤╫╘╣1⁄2╓┌║┼í╛┬φ┐╦╩2╛▌═°í┐_2003.docx]

各位代表：

　　现在，我代表市人民政府，向大会作工作报告，请予审议，并请各位政协委员和其他列席人士提出意见。

　　一、2002年工作回顾

　　2002年是周口发展史上不平凡的一年。一年来，市人民政府在省委、省政府和市委领导下，在市人大、市政协的监督支持下，积极应对加入WTO后的挑战，坚持用市场经济的观点解决前进中遇到的困难和问题，努力探索传统农区的现代化道路，较好地完成了市一届人大二次会议确定的各项任务。

　　国民经济持续增长，质量和效益显著提高。国内生产总值完成414亿元，比上年增长9.5%。其中，第一产业完成152亿元，增长2.4%；第二产业完成161.9亿元，增长14.8%；第三产业完成100亿元，增长12.4%。一、二、三产业由上年的37.9∶38.3∶23.8调整为36.7∶39.1∶24.2，二、三产业所占比重有所上升。社会消费品零售总额完成144.6亿元，增长10.8%。居民消费价格总水平上涨0.8%。财政一般预算收入完成12.8亿元，增长26.6%，其中，市本级一般预算收入完成1.6亿元，增长27.2%。金融机构年末各项存款余额300.9亿元，比年初增加44.8亿元；各项贷款余额325.3亿元，增加28.3亿元。

　　重点项目建设进展顺利，基础设施建设步伐加快。全社会固定资产投资完成104.6亿元，比上年增长16.4%，比全省平均增幅高4.2个百分点。年初确定的38个重点建设项目和26个工业重点项目，完成年度投资30.1亿元，其中，15个项目已经完成，其他一些项目分别进入关键实施阶段和开工准备阶段。新建、改造公路和城区道路2134.5公里，村村通油路率达到70%，每百平方公里公路密度63公里，高于全省平均水平18个百分点。公路改建投资近30亿元。周界高速公路、周淮路等一批公路及北环路、建设路等8条城市道路相继通车；沙河改造一期工程、行政新区和富民经济园区组装正在抓紧进行；市沙南污水处理厂主体工程、市中心血站大楼主体工程、城市电网改造和农网改造一期工程全部竣工，城市功能进一步完善；新增绿地面积40公顷，城市人均公共绿地达到4平方米；以土地收储、出让和路权拍卖为标志，经营城市迈出新步伐；城市规划管理水平有新的提高，小城镇建设进展顺利。

　　工业变点初见成效，新的经济增长点不断壮大。工业增加值完成129.2亿元，增长12.7%。工业用电量突破16亿度，增长19%，创历史最高水平。限额以上工业企业实现利税12.6亿元，增长13.6%。亏损企业亏损额下降61.5%。莲花集团、金丝猴集团、鞋城皮革集团、邦杰集团4家企业迈入省百强企业行列。乡镇企业上缴税金4.69亿元，增长8.8%。工业结构调整步伐加快，全市开发新产品68个。“邦杰火腿肠”被评为中国名牌产品。工业技术改造投资7.3亿元，增长69.1%。郸城金丹乳酸实业有限公司、河南莲花味精集团和黄泛区农场技改项目被列入国债技改项目计划，是我市争取国债技改项目最多的一年。莲花集团技术中心和邦杰集团、郸城金丹乳酸公司技术中心分别被认定为国家级和省级技术中心。邮政、通讯业迅猛发展，邮政业务总量完成1.4亿元，比上年增长10.3%；新增固定电话用户7.3万户、宽带网用户3696户，移动和固定电话用户分别达到41万户和65.7万户，通讯能力和质量全面提高；景区景点建设步伐加快，投资1290万元，开发建设了老子故里、太昊陵、女娲城等景点，旅游综合收入1.16亿元；市场规划建设力度加大，批发市场辐射能力增强；金融、保险、房地产、社区等现代服务业不断发展；非公有制经济增长迅速，在我市经济发展中起着越来越重要的作用。

　　农村税费改革取得阶段性成果，农村经济稳步发展。税费改革工作扎实推进，全市农民亩均负担由129元降至67元，减负率48%，干群关系明显改善，基层政权进一步巩固，县乡财政实力明显增强。种植业结构进一步优化，粮食总产544万吨，粮经比例达到62.6：37.4。农业产业化进程加快，初步形成了小麦、玉米、大豆、棉花和畜产品加工等5大产业链条。围绕龙头企业建成了6个农产品生产基地，16家企业的19个品种被批准为河南省无公害农产品，占全省40%。

　　人民生活水平进一步提高，社会保障能力得到增强。城镇居民人均可支配收入4500元，增长14%；农民人均现金收入1910元，增长5.4%；城乡居民储蓄存款247.8亿元，增长15.4%。社会保障水平有所提高，进再就业中心下岗职工基本生活费、职工养老金、失业保险金发放率均达到100%。城镇登记失业率控制在4%；再就业率达到51%，高于省政府下达目标任务11个百分点。发放低保金2414万元，基本实现了国务院提出的应保尽保的目标。扶贫开发工作取得突破，有4个县分别被国家、省确定为扶贫开发工作重点县，争取扶贫开发资金1.62亿元，社会贫弱群体得到了及时救助，农村特困群体住房难问题初步解决。

　　改革开放稳步推进，经济发展环境有所改善。行政机构改革基本结束，市县两级机构精简10.5%，编制精简20.3%；事业单位改革按照国家统一部署进行了试点。建立现代企业制度工作稳步推进，重点骨干企业法人治理结构逐步完善，中小企业改制面不断扩大。516户企业完成了改制，占应改制企业的80.5%，盘活存量资产16.8亿元。河南辅仁药业集团经营宋河酒厂取得成功。中小学教育体制改革扎实推进，粮食流通体制改革及其他各项改革进一步深化。对外开放步伐加快，利用外资1187万美元，增长85%；完成对外贸易总额1.76亿美元，其中，外贸出口6109万美元，增长35.3%，增幅居全省第六位。清理废止与世贸规则不相适应的文件，加大治乱减负和整顿规范市场经济秩序工作力度，强化审计监督，全面开展了党政领导干部任期经济责任审计。行政服务中心运行良好，受理行政项目24985项，办结24685项，办结率98.8%，较好地发挥了为民服务的窗口示范作用。

　　科教兴市战略深入实施，社会事业全面进步。争取国家、省级科技计划项目72项、科技经费1293万元；新增高科技产品7种、高科技企业4家，2个乡镇跨入省级科技示范乡镇行列；科技进步对经济增长的贡献率达到30%。“两基”教育得到加强，高中教学质量不断提高，普通高招录取人数居全省第二名；改造中小学危房面积8万平方米；新建民办学校19所，在校生达到11.5万人；成立了周口师范学院，填补了周口教育史上无本科院校的空白。全民健身运动广泛开展，竞技体育再创佳绩，获省以上竞技体育比赛奖牌72枚，打破3项省记录。文化市场清理整顿和规范工作继续深化，新闻出版、文化艺术、广播电视事业保持良好发展势头，广场文化、社区文化活动日益活跃。医疗卫生事业有了新发展，医疗水平提高，城乡医疗设施得到改善。计划生育基层基础工作不断加强，实现了省下达的人口与计划生育工作目标。人口自然增长率5.73‰，比上年下降0.56个千分点。耕地保护和土地管理工作扎实推进，土地市场建设和土地资本运营逐步规范。开展严查环境违法行为、遏制污染反弹专项活动，环境质量有所改善。广泛开展“双拥”和军民共建活动，加强军政、军民团结，被省委、省政府、省军区授予“双拥模范城”称号。开发区、气象、外事、侨务、民族、宗教、人防、史志等方面都取得了新的成绩。

　　精神文明和民主法制建设不断加强，社会大局保持稳定。深入开展农村“三个代表”学教活动、“三下乡”活动和群众性精神文明创建活动，不断推进文明城市、文明村镇、文明行业创建工作，广大干部群众精神风貌和城乡面貌有了新的变化。驻村工作扎实有效，对推动农村经济发展、密切干群关系，发挥了积极作用。认真执行人大及其常委会通过的决定和决议，自觉接受人大、政协监督，积极办理人大代表建议和政协委员提案。推行政务公开、村务公开和厂务公开，民主政治建设取得了新成绩。坚持依法治市，加强社会治安综合治理，严打整治斗争取得新成果。廉政建设得到加强，反腐败斗争取得新成效。重视人民群众来信来访，办好市长热线电话，认真解决群众反映的问题，积极化解各种矛盾，维护了社会大局稳定。

　　回顾一年来的实践，我们始终注意把握了以下方面：把发展作为第一要务，坚持市一次党代会确定的思路不动摇，积极探索传统农区现代化的道路；更新观念，用市场经济的办法解决发展中遇到的各种困难和问题；坚持投资拉动经济增长，增强经济发展的后劲；按市场经济要求转变政府职能，继续把优化环境作为经济发展的“一号工程”；正确处理改革、发展、稳定的关系，保持社会大局稳定。

　　各位代表，这些成绩的取得，是省委、省政府和市委正确领导的结果，凝聚了全市广大干部群众的心血和汗水。在此，我代表市人民政府，向奋斗在全市各条战线上的广大工人、农民、知识分子、干部、解放军指战员、武警官兵、政法干警和社会各界人士致以崇高的敬意！向所有为我市经济建设和社会发展做出突出贡献的同志们、朋友们，表示衷心的感谢！

　　在肯定成绩的同时，我们也清醒地看到，我市经济社会发展中还存在不少矛盾和问题。主要是：经济结构仍不合理，经济整体素质和竞争力不强；县域经济发展不平衡，支撑经济持续增长的基础不够稳固；具有核心竞争力的大型企业少，市场竞争力比较弱；农业产业化水平不高，农民增收乏力；地方可用财力偏小，财政刚性支出压力较大；社会保障水平不高，就业和再就业压力增大；整顿和规范市场经济秩序任务仍很艰巨，发展环境有待进一步改善；一些政府部门的工作人员办事效率不高、工作作风不实的问题仍比较突出；一些群众关心的热点、难点问题依然存在。对此，我们一定高度重视，在今后的工作中着力加以解决。

　　二、以全面建设小康社会总揽全局，切实做好2003年各项工作

　　今年是全面贯彻党的十六大精神的第一年，中央提出，从现在到2020年，要实现全面建设小康社会的奋斗目标，达到人均国内生产总值3000美元。目前，全国人均国内生产总值已达800美元，全省600美元，我市仅为400美元。以2000年为基数计算，全国翻两番，周口必须翻三番，发展任务非常艰巨。我们必须树立强烈的忧患意识和危机意识，努力做到发展要有新思路，改革要有新突破，开放要有新局面，各项工作要有新举措，充分估计可能出现的各种困难，充分利用一切有利条件，审时度势，趋利避害，尽力发挥比较优势，积极寻求后发优势，集全市之智，纳各方之策，举全民之力，聚精会神搞建设，一心一意谋发展，实现经济与社会事业协调发展，物质文明与精神文明共同进步。

　　今年政府工作的总体要求是：以邓小平理论和“三个代表”重要思想为指导，认真贯彻党的十六大精神，紧紧围绕全面建设小康社会的奋斗目标，进一步解放思想，加快工业化、城镇化和农业产业化进程，加大对外开放力度，加强基础设施和重点项目建设，不断调整和优化经济结构，大力推进新型工业化，积极发展农业和农村经济，加快农村富余劳动力向非农产业和城镇转移，放手发展非公有制经济，深化改革，优化环境，保持稳定，搞好社会主义政治文明和精神文明建设，切实做好就业和再就业工作，完善社会保障体系，促进国民经济持续快速发展和社会全面进步，不断提高人民生活水平。

　　今年经济社会发展的主要预期目标是：国内生产总值增长9%，力争达到10%。其中，第一产业增长4.5%，第二产业增长12.5%，第三产业增长10%；全社会固定资产投资增长15%；社会消费品零售总额增长10%；外贸出口增长12%，实际利用外资增长15%；地方财政收入与国内生产总值同步增长；城镇居民人均可支配收入增长6%，农民人均纯收入增长5%；人口自然增长率控制在6.5‰以内；城镇登记失业率控制在4%以内。

　　为实现上述目标，今年，市人民政府重点抓好以下十二个方面的工作：

　　(一)加快工业结构调整，走新型工业化道路

　　运用高新技术和先进适用技术改造传统产业，有选择地发展高新技术产业，优化工业结构，促进工业升级，走新型工业化道路。继续发展电力、交通、通讯等基础产业，营造工业发展的平台。大力发展近农工业，培育和壮大龙头企业。以莲花味精集团为依托拉长粮食加工链条，以邦杰集团为依托拉长肉制品生产加工链条，以益海粮油工业有限公司为依托拉长油料加工链条，以鞋城皮革集团为依托拉长皮革加工链条，以资源优势和规模优势组建跨县域的棉纺织集团，形成周口特色的产业链条。有重点地选择邦杰集团、金丝猴集团、鞋城皮革集团等有条件的企业，帮助和策划上市融资。鼓励企业应用信息技术，提高信息化水平，以信息化带动工业化。充分利用人力资源，大力发展劳动密集型产业，形成劳动密集型产业与资金技术密集型产业优势互补、协调发展的产业格局。继续抓好去年确定的工业重点项目中未完工项目的建设，选择好新上项目，按照当年竣工投产、续建、新开工和前期工作四个梯队，依次推进，促进工业经济快速发展。建立和完善企业自主创新机制，使企业成为技术进步和创新的主体。加大对专、新、特、精中小企业的支持力度，加强中小企业服务体系建设，成立中小企业融资担保服务中心，引导中小企业走科技型和外向型发展道路。

　　(二)加快城乡结构调整，不断提高城镇化水平

　　围绕“缩短战线，搞好组装，完善功能，提高品位，强化管理”的基本思路，在抓好新区建设的同时，重视旧城改造。抓好续建工程建设，继续实施沙河改造一期工程，加快行政新区、富民经济园区、开发区的建设和周(口)商(水)一体化步伐，延伸大庆路向南至行政新区，中州路、大庆路、八一路向北至北环路，推进水、电、气、暖等城市基础设施建设。积极探索经营城市的路子，加快建立投资主体多元化、投资方式多样化、项目实施市场化的新机制，多渠道筹措城市建设资金。采取股份制、特许经营、转让经营权等多种运作方式，对有条件的城市基础设施实行市场化运营。建立和完善土地收购储备和开发利用制度，制订土地垄断经营的近、远期规划，提高城市土地收益。加大城市管理力度，落实目标管理责任制。以创建文明城市为载体，做好绿化、亮化、美化工作，实现城郊绿化和通道绿化人格化，注重丰富和提升市区道路绿化的品种和档次，达到人与自然的和谐，提高城市品位。

　　加快农村城镇化进程，推动乡镇企业、农业产业化经营和集贸市场向县城和小城镇集中，重视增强城镇功能，形成产业支撑，逐步建成以县城为主体、以重点乡镇为补充的规模适度、布局合理、经济繁荣、特色鲜明的小城镇群体，着重抓好付井、逍遥、四通、玄武、秣陵五镇建设。按照“统一规划，量力而行，讲究实效，农民自愿”的原则，继续抓好县乡公路建设，逐步实现村村通油路的目标。

　　(三)加快农业结构调整，全面繁荣农村经济

　　全面奔小康，重点在农村。作为农业大区，必须统筹城乡经济社会发展，更多地关注农村，关心农民，支持农业，把解决好农业、农村、农民问题作为经济工作的重中之重，放在更加突出的位置，努力开创农业和农村工作的新局面。切实转变农业和农村工作指导思想和工作方法，用工业理念发展农业。积极推进传统农业向现代农业转变，进一步调整和优化农业种植结构，促进大宗农产品生产优质化，特色农产品生产多样化和规模化。围绕培育具有地域优势的特色产业，加快优质农产品、林果业产品和畜牧养殖基地建设，大力发展优质、高产、高效、生态安全农产品，扩大精细蔬菜、瓜果、花卉、中药材的产业规模。建立健全农产品质量标准体系和检验检测体系，保证农产品质量安全。发展标准农业、绿色农业、订单农业和创汇农业，提高农产品的市场竞争力。积极推进农业产业化经营，重点扶持莲花、邦杰、益海、鞋城皮革等一批辐射面广、带动力强的农产品加工、储运、保鲜龙头企业。充分引导和发挥各类行业协会和农村经济合作组织在开拓市场和建设现代农业中的作用，解决农村经济组织缺位问题。通过体制创新、技术改造、布局优化、产业升级，提高乡镇企业竞争力。抓住国家用于农业和农村的投入向粮食主产区倾斜的机遇，争取更多的政策支持。发挥金融支持农村经济增长的重要作用，充分利用扶贫开发资金、国债资金和金融小额信贷资金，加快农村基础设施建设。搞好新时期农业综合开发和扶贫开发，以扶贫重点村建设为突破口，抓好政策扶贫、项目扶贫、信贷扶贫、科技扶贫和社会扶贫工作。大力发展“打工经济”，推进农村富余劳动力转移，拓展农民生存发展空间，增加农民收入。

　　(四)搞好重点项目建设，扩大固定资产投资

　　继续实施项目带动战略，全市安排重点项目38个，计划总投资75亿元。其中，竣工项目15个，今年完成投资13.8亿元；续建项目7个，今年完成投资6.4亿元；新开工项目16个。市本级集中精力运作或帮助大企业运作投资1亿元以上的大项目，不断培育支撑全市经济增长的新骨干；县市区注意发挥比较优势，帮助企业重点运作一批5000万元以上的投资项目，着力培育经济支柱；各类中小企业不断拓展新项目，壮大竞争实力。继续加大项目工作力度，争取有更多的项目进入国家国债贴息、双高一优和省结构调整政策支持的盘子。大力拓宽融资渠道，在积极争取国家政策性资金和国有银行支持的同时，千方百计寻找合作伙伴，与国内外知名的大公司合资合作。进一步加强对项目工作的领导，实行主要领导负责制，把进度、责任落到实处。完成周西220千伏变电站、农网二期改造工程和隆达发电有限公司2×13.5万千瓦机组技改工程等电力基本建设项目。完善城市道路交通网络，抓好地铁升级改造、益海工业园区和邦杰工业园区等重点项目建设。

　　(五)把对外开放作为重点，全面提高对外开放水平

　　经过撤地设市两年来的建设和发展，城市框架初具雏形，交通网络逐步形成，工业化步伐加快，相伴而来的缺资金、技术、项目和人才的问题日益突出，仅靠我们自己的力量去搞建设，推进城市化、工业化和农业产业化，是不可能的，也是不现实的。在经济全球化的大背景下，我们必须以开放促发展，实施“引进来”与“走出去”并举的开放战略，把利用外资与对外投资、扩大出口与优化进口结合起来。抢抓发达国家产业资本向中国转移、沿海产业资本向内地转移的机遇，加强与境外华人商会、沿海发达地区、改革开放先进地区、周边地区的联系，扎实做好引资工作。创新招商方式，开展高层次、小团组、专业化、针对性的招商活动，积极探索网上招商、委托中介机构有偿代理招商等办法，扩大引资领域。把企业作为招商引资的主体，鼓励支持莲花集团、邦杰集团、鞋城皮革集团等基础条件较好的企业，主动与世界著名的跨国公司对接，争取合资合作。切实办好现有外资企业，发挥示范效应，以商招商、以外引外。把引进市外资金、技术、人才、先进的管理理念、管理方式作为对外开放的重要方面，实行招商引资目标责任制。强化亲商观念，完善相关政策措施，建立投资环境综合评价体系，实行投资环境“一票否决”，从整体上优化投资环境。鼓励有条件的企业开展境外加工贸易或到境外承包工程、投资办厂，发挥比较优势，参与国际分工。进一步深化外贸体制改革，不断扩大外贸经营主体，积极扶持更多的企业拓展自营进出口业务。加快培育一支善于跨国经营的外贸人才队伍。继续扩大对外劳务输出规模，不断提高输出劳务的技术档次，逐步实现由体力型向技术型的转变。认真研究开拓西部市场，鼓励有条件的企业到西部发展，实现低成本扩张，努力扩大我市产品在西部市场的占有份额。

　　(六)加快所有制结构调整，放手发展非公有制经济

　　毫不动摇地鼓励、支持和引导非公有制经济发展，坚决冲破一切妨碍非公有制经济发展的思想观念，坚决改变一切束缚非公有制经济发展的做法和规定，坚决革除一切影响非公有制经济发展的体制弊端。充分发挥个体私营等非公有制经济在促进经济增长、扩大就业和活跃市场等方面的重要作用，支持个体私营企业全方位进入一、二、三产业和社会事业发展领域。引导个体私营经济向科技型、就业型、创汇型、社区服务型发展，推动个体私营企业参与国有工商企业改制。建立重点私营企业联系制度和定期协调会议制度，组织实施私营经济扶优扶强工程，研究解决私营经济发展中的重大问题。完善个体私营企业贷款担保体系。加强劳动合同管理，推动公证、职业介绍、信息咨询等中介组织为个体私营经济提供规范服务，切实维护个体私营企业的合法权益。认真落实政策和法律法规，使不同所有制企业在市场准入、土地使用、贷款担保等方面享受平等待遇，为非公有制经济发展创造平等的竞争环境，努力使非公有制经济增加值占国内生产总值的比重有较大提高。

　　(七)加强财税金融工作，支持扩大内需

　　采取积极的财政措施，大力加强财源建设，下决心壮大市本级财政。大力发展工业主体税源，积极拓展新兴后续财源。在主要依靠自身努力不断壮大财政实力的同时，积极争取上级财政的支持。高度重视乡镇财政建设，加强指导，落实责任，通过经济增长点和财税支撑点的培植和管理水平的提高，促进乡镇财政状况逐步好转。按照“保工资、保运转、保稳定、兼顾重点”的原则，合理安排财政支出。以机构改革为契机，减人减事减支出，建立公共财政的基本框架。坚持有所为有所不为，集中财力支持重点项目建设，保障全局性、长远性重大目标任务的完成。继续贯彻“加强征管、堵塞漏洞、惩治腐败、清缴欠税”的方针，加强财税收入管理，坚持依法办事，依率计征。严厉打击各种偷税、抗税、骗税和逃税行为，大力清理压缩欠税，保证税收足额均衡入库。

　　落实扩大内需和调整经济结构的各项金融措施，加快保险业的改革与发展，大力发展证券市场，充分发挥金融对经济发展的支持作用。积极向银行推荐项目，支持重点企业的发展和重点工程建设。扩大信贷投入，增加对企业技术改造、农业经济结构调整、信息化建设和民营科技企业的资金支持。加强金融监管，整顿和规范金融秩序，取缔非法金融活动，开展信用村户建设，防范和化解金融风险，创建金融安全区。

　　(八)大力发展第三产业，积极培育新的经济增长点

　　重点发展以服务业为主的第三产业，千方百计推进服务业的市场化、产业化和社会化进程，使之逐步成为扩大就业的渠道、促进消费的主体、经济增长的载体和中心城市的重要支撑产业。围绕中心城市建设，重点培育一批有较强辐射力的专业市场，逐步形成以市带县、以城促乡的市场网络。进一步规范完善荷花市场、中州商城、铁路建材城、蔬菜批发市场、种子农药市场，筹建粮食交易市场、农贸市场等专业市场，积极推行连锁经营、代理经营、物流配送等现代流通方式，增强流通辐射功能。面向城乡居民消费，积极发展房地产业。发展经济适用住房和廉租房，逐步满足低收入群众住房需求。借助新区组装、旧城改造，努力扩大住房消费信贷规模，鼓励和引导住房消费。积极发展和规范房地产中介市场，切实维护房地产商和居民的合法权益。建立完善包括物业管理、卫生保健、家政服务等在内的社区服务体系，引导社区服务业快速健康发展。加大关帝庙、太昊陵、龙湖、老子故里等景区的基础设施建设，搞好旅游资源开发利用，加快旅游业发展。积极发展信息、咨询、技术服务、资产评估等市场中介组织，引导文化娱乐、教育培训、体育健身等产业发展，满足服务消费需求。

　　(九)继续完善社会保障体系，千方百计扩大就业

　　加快社会保障体系建设，加大财政对社会保障工作的支持力度，做好“两个确保”和“三条保障线”的衔接工作。安排好困难企业特困职工的生产生活，及时发现和解决存在的问题。落实城市居民最低生活保障制度，把所有符合条件的贫困居民全部纳入最低生活保障范围，做到应保尽保。建立和完善对特困人群的救助制度，帮助他们解决实际困难。巩固和扩大城镇养老、医疗和失业保险等社会保险覆盖面，强化社会保险费的征缴和支出管理。有条件的县市区，探索建立农村养老、医疗保险和最低生活保障制度。做好优抚安置工作，加大社会救济力度，提倡社会互助，发展慈善事业，推进各类社会福利事业的社会化进程。

　　千方百计抓好就业和再就业工作，引导全社会转变就业观念，把扩大就业与经济结构调整结合起来，通过发展非公有制经济、劳动密集型产业，拓宽就业渠道，创造就业机会。大力开展职业技术培训，推行灵活多样的就业形式，鼓励自谋职业和自主创业。加大对劳动力市场建设的投入，加快市、区、街道三级再就业网络建设。积极搭建促进就业和下岗职工再就业的服务平台，建立就业和再就业援助制度，为就业困难对象提供专门的帮助和服务，维护好社会稳定大局。

　　(十)继续整顿和规范市场经济秩序，进一步优化经济发展环境

　　把集中整治与建立长效机制结合起来，采取集中行动，坚决制止对企业进行乱摊派、乱收费、乱罚款、乱检查。深入开展打假打诈联合行动，加强知识产权保护，严厉打击制售假冒伪劣商品等违法犯罪活动。继续整顿和规范建筑市场、文化市场和税收征管秩序，坚持不懈地打击传销和走私违法犯罪活动。打破地方保护和行业垄断，形成统一、开放、竞争有序的市场。重视信用制度建设，提高企业和个人的诚信意识，在全社会营造诚实守信、公平竞争的氛围，打造诚信周口。行政执法部门要增强服务意识，规范执法行为，严禁以罚代管、以罚代法。

　　严肃财经纪律，加强对企业会计信息质量的监督。规范市场中介组织的行为，发挥其维护市场秩序的积极作用。切实加强安全生产和安全监察，强化安全生产责任制，严格执行重特大事故行政责任追究的规定，遏制重特大事故的发生。

　　(十一)深化改革，为发展提供强大动力

　　集中力量抓好重点企业和企业集团的规范运行，不断完善法人治理结构，切实转换经营机制，实现股权多元化，加快培育一批主业突出、具有核心竞争力的大企业和企业集团。认真解决改制企业的遗留问题，扎实做好规范完善和巩固提高工作。建立和完善国有中小企业退出机制，采取联合、兼并、破产等多种资产重组方式，使国有资本基本退出竞争性行业，从整体上搞活中小企业。深化企业内部改革，加强企业现代化管理，狠抓扭亏增盈工作，努力提高企业经济效益。

　　稳定并不断完善以家庭承包经营为基础、统分结合的双层经营体制，有条件的县市区可按照依法、自愿、有偿的原则进行土地承包经营权流转，逐步发展规模经营。发展农村中介服务组织，壮大农村经纪人队伍，培育和完善农村市场体系。推进粮食流通体制改革，逐步实现粮食购销市场化。进一步巩固税费改革成果，落实各项农村税费改革政策及配套措施，坚决制止各种形式的向农民乱摊派、乱收费行为，严防农民负担反弹，确保税费改革取得圆满成功。积极探索对农业和农民实行补贴的有效办法，加大对农业的扶持力度，保护好农民的利益。

　　积极推进行政体制改革，进一步完善行政服务中心功能，坚持“一个窗口对外”，杜绝两头受理，积极探索简洁高效的服务途径。继续推进政府机构改革，实现机构和编制的法定化。按照政事分开原则，合理划分党政机关与事业单位职责，改革事业单位管理体制。进一步清理、规范行政审批事项和收费项目，对不符合政企分开和政事分开原则的行政审批，坚决予以取消。强化行政管理监督机制，加大监督与查处力度，设立“160”举报投诉电话。真正把政府职能转到经济调节、市场监管、社会管理和公共服务上来，促进政府职能和服务方式的根本性转变。

　　(十二)加快发展各项社会事业，加强社会主义精神文明和民主法制建设

　　坚持“三为主”方针，稳定低生育水平，认真落实人口与计划生育目标管理责任制和一票否决权，重点做好农村、城郊结合部和流动人口的计划生育工作，确保人口自然增长率低于全省平均水平；合理开发、节约使用和保护土地资源，完善资源有偿使用机制，提高土地利用效率；坚持生态环境保护和污染防治并重，突出水污染防治，加强城市空气质量监测管理，严格执行建设项目环境评价制度和“三同时”制度，使城乡环境质量明显改善。

　　加强科研项目库建设，组织申报国家级、省级高新技术产业化项目和中小企业技术创新项目。积极开展科技知识宣传和普及，努力提高全社会的科技意识。加大中小学危房改造工程的实施力度，巩固“两基”成果，提高义务教育办学水平，大力推进素质教育，扩大普通高中招生规模。优化教育资源配置，大力支持周口职业技术学院建设，积极发展成人教育和其他继续教育，鼓励自学成才。支持社会力量办学，抓好中国人民大学豫东附属中学的筹建。逐步解决市区适龄儿童入学难和学生大班额问题。继续加大对教育的投入，确保教师工资按时足额发放。认真落实文化发展政策，切实加强新闻出版管理和文物保护工作，加快文化馆、博物馆和图书馆“三馆”筹建步伐，进一步繁荣文化艺术。开工建设周口广播电视中心，进一步提高广播电视覆盖率。加强农村卫生工作，积极探索农村新型合作医疗的路子，重点做好公共卫生和以艾滋病为重点的重大疫病防治工作；全面落实《全民健身计划纲要》，发展社区体育，不断提高竞技体育水平。

　　重视统计工作，提高对经济运行的监测、预测水平。搞好国防教育，增强全民国防意识。广泛开展军民共建和“三好”服务活动，进一步密切军政军民关系。做好民兵预备役工作，发挥民兵预备役人员在两个文明建设中的作用。认真贯彻落实党的民族宗教政策，依法管理民族宗教事务，积极引导宗教与社会主义社会相适应。关心妇女儿童和老龄工作，依法维护老年人、妇女儿童和残疾人的合法权益。继续做好物价、地震、人防、外事、侨务等工作，促进社会事业全面发展。

　　认真实施《公民道德建设纲要》，不断提高全社会的道德水平和道德境界。继续抓好科技、文化、卫生“三下乡”，丰富广大农民的精神文化生活。加强基层民主建设，完善居民自治体制。继续推行政务公开、村务公开、厂务公开，切实保障群众的民主权益。认真执行人民代表大会及其常委会的决议和决定，自觉接受人民代表大会及其常委会和人民政协的监督。重视支持人民政协政治协商和参政议政，加强与各民主党派、工商联、无党派人士及工、青、妇等群众团体的联系和沟通，发挥他们联系群众的桥梁和纽带作用。

　　认真实施“四五”普法和依法治市规划，深入开展普法教育，提高公民法律素质。加强政府法制建设，提高依法决策、依法行政水平。加强社会治安综合治理，实施“农村平安工程”，继续贯彻严打方针，依法从重从快打击严重刑事犯罪，铲除严重危害社会的各种犯罪团伙和黑恶势力，继续开展与“法轮功”等邪教组织的斗争。高度重视人民群众来信来访工作，及时化解各种矛盾和纠纷，确保社会大局稳定。

　　三、实践“三个代表”重要思想，建设廉洁、勤政、务实、高效的政府

　　全面完成今年各项目标任务，实现经济快速发展，对政府工作提出了新的要求。我们要认真实践“三个代表”重要思想，把发展作为第一要务，保持与时俱进的精神状态，全神贯注、兢兢业业地做好各项工作，建设一个“行为规范、运转协调、公正透明、廉洁高效”的政府。

　　建设学习型政府。在世界多极化、经济全球化、服务社会化的新背景下，建设学习型政府，是适应新形势、新任务的必然要求，是迎接新挑战的必然选择。从政府领导层做起，倡导多读书、多思考、多实践，深刻领会邓小平理论和“三个代表”重要思想，学会用发展的、联系的、辩证的观点观察、分析、解决问题。认真学习政治、经济、文化、科技、法律等方面的最新知识和理论，切实增强各级干部特别是领导干部工作的适应性、预见性、主动性和创造性，提高行政水平。

　　建设务实为民的政府。从实际出发，全面了解和把握市情，改进工作作风，强化落实机制。坚决克服形式主义、官僚主义，说实话，重实干，求实效。所有政府工作人员务必继续保持谦虚谨慎、不骄不躁的作风，务必继续保持艰苦奋斗的作风，时刻牢记我们的职责和使命，办任何事情都要注重实效。对市一次党代会和政府确定的目标任务，都要排除万难，一抓到底，保持工作的连续性，一任接着一任干，一张蓝图绘到底，做到工作任务、责任人、时间表和配套条件四落实。对经过人大审议通过后的本次《政府工作报告》提出的各项任务，会后都要量化分解，建立严格的考核制度，加强督查，确保各项工作落到实处。要牢固树立立党为公、执政为民的权力观，保持同人民群众的血肉联系，全心全意为人民服务。坚持深入基层、深入群众，认真倾听群众呼声，关心群众安危冷暖，做到权为民所用、情为民所系、利为民所谋，带领群众创造幸福生活。

　　建设廉洁高效的政府。进一步抓好领导干部廉洁自律、查处大案要案、纠正部门和行业不正之风三项重点工作。坚持标本兼治、综合治理，从源头上预防和治理腐败。继续实行领导干部任期经济责任审计制度，严格审计监督。实行多种形式的述职述廉制度，健全重大事项报告制度、质询制度和民主评议制度。把反腐倡廉同改革开放和经济建设的重大措施结合起来，使党风廉正建设和反腐败工作同改革开放和经济建设相互促进、协调发展。加强效能建设，全面推行办文办事限时制，减少繁文缛节，简化办事程序，提高办事效率。进一步强化全局观念，加强政府部门之间的密切协作，更好地发挥政府的整体效能。

　　各位代表，我们正处在全面建设小康社会的重要历史时期，今年的任务光荣而艰巨。让我们紧密地团结在以胡锦涛同志为总书记的党中央周围，在市委的领导下，坚定信心，励精图治，同心同德，埋头苦干，共同谱写我市改革开放和现代化建设事业更加辉煌壮丽的新篇章！
